# Integrated proteomics and metabolomics analysis of rice leaves in response to rice straw return

**DOI:** 10.3389/fpls.2022.997557

**Published:** 2022-09-13

**Authors:** Shuangshuang Yan, Shengdan Gong, Kexin Sun, Jinwang Li, Hongming Zhang, Jinsheng Fan, Zhenping Gong, Zhongxue Zhang, Chao Yan

**Affiliations:** ^1^College of Agriculture, Northeast Agricultural University, Harbin, China; ^2^Institute of Forage and Grassland Sciences, Heilongjiang Academy of Agricultural Sciences, Harbin, China; ^3^College of Water Conservancy and Civil Engineering, Northeast Agricultural University, Harbin, China

**Keywords:** rice straw return, rice, plant growth, leaf, proteomics, metabolomics

## Abstract

Straw return is crucial for the sustainable development of rice planting, but no consistent results were observed for the effect of straw return on rice growth. To investigate the response of rice leaves to rice straw return in Northeast China, two treatments were set, no straw return (S0) and rice straw return (SR). We analyzed the physiological index of rice leaves and measured differentially expressed proteins (DEPs) and differentially expressed metabolites (DEMs) levels in rice leaves by the use of proteomics and metabolomics approaches. The results showed that, compared with the S0 treatment, the SR treatment significantly decreased the dry weight of rice plants and non-structural carbohydrate contents and destroyed the chloroplast ultrastructure. In rice leaves of SR treatment, 329 DEPs were upregulated, 303 DEPs were downregulated, 44 DEMs were upregulated, and 71 DEMs were downregulated. These DEPs were mainly involved in photosynthesis and oxidative phosphorylation, and DEMs were mainly involved in alpha-linolenic acid metabolism, galactose metabolism, glycerophospholipid metabolism, pentose and gluconic acid metabolism, and other metabolic pathways. Rice straw return promoted the accumulation of scavenging substances of active oxygen and osmotic adjustment substances, such as glutathione, organic acids, amino acids, and other substances. The SR treatment reduced the photosynthetic capacity and energy production of carbon metabolism, inhibiting the growth of rice plants, while the increase of metabolites involved in defense against abiotic stress enhanced the adaptability of rice plants to straw return stress.

## Introduction

Rice is an important food crop species, and a large amount of rice straw is produced during rice cultivation. Straw return avoids the environmental pollution caused by straw burning and plays an important role in the sustainability of cropping systems. Straw return increases soil nutrients, organic carbon content, and the relative abundance of the microbial community, improves soil enzyme activity and soil structure (Yao et al., 2015; Li et al., 2019; Yan et al., 2020), and significantly affects soil pH, cation exchange capacity and electrical conductivity (Blanco-Canqui and Lal, 2009). Warm conditions promote straw decomposition and nutrient release (Yadvinder et al., 2005). In a subtropical monsoon climate, compared with no straw return, straw return increases rice and wheat yields in a rice-wheat rotation (Hu et al., 2016; Yuan et al., 2021), and also increases rice yield in a double rice cropping system (Zhang et al., 2017). However, straw return decreases the yield of direct-seeded rice, the reasons for the yield decrease are currently unknown (Yang et al., 2020). In cold waterlogged paddy soils of North China, straw return significantly reduces rice yield and tillers number (Cui et al., 2017). Straw return typically results in microbial N immobilization, especially cereal straw with a high C/N ratio, and a temporary decrease in plant-available N (Thuy et al., 2008; Xu et al., 2010). Straw return enhances more reducing conditions in submerged soil (Tanji et al., 2003), leading to the accumulation of organic acids and sulfide in the soil, which would inhibit the growth of rice and reduce rice yield (Gao et al., 2004; Shan et al., 2008). There are no consistent results observed about the effect of straw return on crop yield, which is affected by many factors (Huang et al., 2013; Pittelkow et al., 2015).

Leaves are important organs of plants that use light energy to convert CO_2_ into sucrose, and their photosynthetic capacity is directly related to crop yield. To adapt to severe environment, plants have developed complex, well-coordinated molecular and metabolic networks to regulate growth, photosynthesis, osmotic maintenance, and carbohydrate homeostasis (Saddhe et al., 2021). Photosynthesis is the most sensitive mechanism to abiotic stress, and carbon assimilation and primary metabolism are greatly affected when plants are exposed to adverse environmental conditions. Sugars, glycols, and amino acids are the most important metabolites, and their concentrations in plant tissues are affected by environmental stress, usually as a result of impaired CO_2_ assimilation processes and complex regulatory networks (Valerio et al., 2011; Krasensky and Jonak, 2012). Sugars contribute directly or indirectly to antioxidative mechanisms (Keunen et al., 2013). Plants induce the synthesis of osmotic regulators such as soluble sugars and amino acids, and maintain cell turgor through osmotic regulation (Arbona et al., 2003).

Proteomics and metabolomics are used to analyze the response of plants to abiotic stress (Kang et al., 2010; Arbona et al., 2013). Dehydration induced damage to the chloroplast ultrastructure of rice seedling leaves, increased free amino acid abundance, and the DEPs were presumably involved in chloroplast energy metabolism, photosynthesis, and defense response (Gayen et al., 2019). At the flowering stage and milk stage, the levels of defense-related proteins and antioxidases are increased in rice flag leaves under drought stress, the ROS scavenging system is active, photosynthesis and CO_2_ assimilation are damaged, and redox imbalance occurs (Wang et al., 2017). Under salt stress conditions, the DEPs in rice shoots are mainly involved in photosynthesis, antioxidant, and oxidative phosphorylation at the four-leaf stage (Xu et al., 2015), while DEPs are mainly involved in defense to oxidative stresses, metabolisms, photosynthesis, protein synthesis and processing, signal transduction at early vegetative stage of rice (Ghaffari et al., 2014). Combined proteomics and metabolomics analysis of rice plants is helpful to reveal the key metabolic and regulatory pathways of the rice response to abiotic stress (Oikawa et al., 2008; Kim et al., 2014a), but there is still little research about the effect of rice straw return on rice leaves.

Heilongjiang Province, located in Northeast China, is the largest japonica rice production area, and the decomposition of rice straw is slow due to the cold climate (Yan et al., 2019). Most of rice straw is burned *in situ*, straw burning seriously pollutes the environment (Qu et al., 2012), leads to the loss of soil organic matter and nutrients, and reduces soil microbial activities (Kumar et al., 2019). The nitrogen and phosphorous contents in the soil solution are reduced with rice straw return (Yan et al., 2015, 2018), which affects rice growth. To study the effect of rice straw return on rice growth, we conducted a study in Northeast China and applied two treatments: no straw return and rice straw return. In this study, the physiological indexes of rice leaves and chloroplast ultrastructure were observed, and the changes in DEP and DEM levels were investigated through the combined proteomics and metabolomics analysis of rice leaves. This study aimed to reveal the metabolic pathways of rice leaves in response to rice straw return, which could provide reference information for clarifying the physiological mechanism through which rice plants resist straw return stress.

## Materials and methods

### Experimental design

This experiment was conducted at the experimental station of Northeast Agricultural University. The station is located in Harbin, Heilongjiang Province (126°43′E, 45°44′N), the region of which has cold temperate continental climate. The annual precipitation is 500–550 mm and is mainly concentrated from June to September. The cumulated active (≥10°C) air temperature was more than 2,700°C. Rice crops are grown once a year.

A pot experiment was conducted in 2020. Plots with a diameter of 30 cm and a height of 35 cm were filled with 20 kg of soil. The soil used was Mollisol taken from plow-layer soil. The basic soil physicochemical properties are shown in Supplementary Table 1. The experiment included two treatments: no straw return (S0) and rice straw return (SR). The amount of rice straw returned was 80 g pot^–1^ (12,500 kg ha^–1^) according to Yan et al. (2020), and each treatment was replicated 20 times. Air-dried rice straw was cut into approximately 5 cm pieces. On May 16th, the rice straw was evenly mixed with the soil, put into pots and soaked. On May 23rd, 30-day-old seedlings were transplanted, with 3 seedlings hole^–1^ and 3 holes pot^–1^. Before transplanting, basal fertilizer-1.06 g urea (150 kg ha^–1^), 1.11 g Ca(H_2_PO_4_)_2_ (157 kg ha^–1^) and 0.71 g K_2_SO_4_ (100 kg ha^–1^)-was applied to each pot. Rice samples displaying the same growth were collected from each treatment 30 days after rice transplanting.

### Sampling methods

The rice plants were sampled and divided into shoots and roots. The roots were washed with distilled water, and the dry weights of the shoots and roots were determined after oven drying at 80°C to a constant weight. The fully expanded uppermost leaves were harvested for transmission electron microscopy (TEM), and the middle sections of the rice leaves were sliced into 1 mm × 3 mm pieces and fixed in 2.5% glutaraldehyde at 4°C. The fully expanded leaves from the upper part of rice plants were collected between 10:00 a.m. and 12:00 p.m., rinsed with phosphate-buffered solution, drained and wrapped in aluminum foil. The samples were frozen in liquid nitrogen and then stored at −80°C for non-structural carbohydrate content, proteomic and metabolomic analyses. The tests were repeated three times for the proteomics analysis and six times for the metabolomics analysis.

### Determination of non-structural carbohydrate content

The non-structural carbohydrate content was determined by enzyme-linked immunosorbent assays (ELISAs). A 0.1 g fresh rice leaf sample was used to analyze the starch, sucrose and soluble sugar contents using plates (Shanghai Enzyme Linked Biotechnology Co., Ltd., Shanghai, China), with five replications per treatment.

### Transmission electron microscopy

The 1 mm × 3 mm pieces were post-fixed in 1% osmic acid, and the samples were dehydrated in an ethanol series after rinsing in phosphate buffer and dehydrated with acetone. The dehydrated tissues were embedded in Epon 812 resin, sliced into ultrathin sections using an LKB V ultramicrotome (LKB, Stockholm, Sweden), and stained with both uranyl acetate and lead citrate. Chloroplast ultrastructure was observed and imaged using an H-600 transmission electron microscope (Hitachi, Tokyo, Japan) (Yamane et al., 2003a).

### Proteomics analysis

#### Protein extraction and tandem mass tags labeling

Rice tissues were ground in liquid nitrogen. We used the phenol extraction method. Afterward, using the bicinchoninic acid assay (BCA) protein assay, the protein concentration of the supernatant was determined, and then 100 μg of protein per condition was transferred into a new tube, after which the final volume was brought to 100 μL. Then, 5 μL of 200 mM DTT was added, and the sample was incubated at 50°C for 1 h. Next, 5 μL of 700 mM iodoacetamide was added to the sample and incubated for 30 min in the dark at room temperature. The proteins were digested with sequence-grade modified trypsin (Promega, Madison, WI, United States).

The protein concentration of the supernatant was determined with the BCA protein assay. After the peptide solution was freeze dried and dissolved in 100 μL of ultrapure water, the final concentration of triethylammonium bicarbonate (TEAB) was approximately 100 mM. The tandem mass tags (TMT) labeling reagent was subsequently equilibrated at room temperature. The labeling reagent was added to the corresponding peptide solution according to the labeling information table, and different samples were labeled with isotopes of different sizes. The labeled samples were mixed into one sample, desalted using a C18 SPE column (Sep-Pak C18, Waters, Milford, MA, United States), and, then, vacuum freeze dried.

#### High pH reversed-phase separation

Mobile phase A consisted of 10 mM ammonium formate in water (pH 10.0), adjusted with ammonium hydroxide, and mobile phase B consisted of 10 mM ammonium formate in 90% acetonitrile (ACN) (pH 10.0) and adjusted with ammonium hydroxide. Analytes were separated by using an ultra performance liquid chromatography (UPLC) system (Waters Corporation, Milford, MA, United States) connected to a reversed-phase column (BEH C18 column, 2.1 mm × 150 mm, 1.7 μm, 300 Å; Waters Corporation, Milford, MA, United States), and the loading amount was 50 μL. The flow rate was 250 μL min^–1^, and UV detection was performed at 215 nm. According to the chromatographic peak pattern, 12 fractions were collected and vacuum freeze dried for the next step.

#### Low pH nano-high performance liquid chromatography-tandem mass spectrometry analysis

The composition of liquid C was water and 0.1% formic acid (FA); the composition of D was ACN and 0.1% FA. The fractions were separated with nano-LC, the chromatographic column type was C18, and the flow rate was 300 nL min^–1^. Each fraction was dissolved in 40 μL of liquid C, and the loading amount was 4 μL; the samples were analyzed *via* liquid chromatography tandem mass spectrometry (LC-MS).

An Orbitrap Fusion mass spectrometer was used for each sample, with data-dependent mode automatically switched between MS and MS/MS. The mass spectrometer was operated in positive ion mode, and the primary scanning range was 350–1,550 Da, with a mass resolution of 120,000 at m/z 200. The secondary scanning range was automatically selected depending on the mass-to-charge ratio of the primary precursor ions. The capillary temperature was 300°C, the ion tube voltage was 2,000 V, and the fragmentation mode was higher energy collisional dissociation (HCD).

#### Database searching and quantitative data analysis

The mass spectra were filtered using Proteome Discoverer (PD) software (version 1.4.0.288, Thermo Fisher Scientific Inc., MA, United States). The spectrum extracted by PD was searched by Mascot (version 2.3.2, Matrix Science Ltd., London, UK). After the search, quantitative analysis was performed *via* the PD software according to the search results of Mascot and the spectrum after the first step of screening.

### Metabolomics analysis

Metabolite extraction: 50 mg of sample was weighed, after which 1,000 μL of extraction solution [methanol: acetonitrile: water = 2: 2: 1 (V/V), including 1 μg mL^–1^ internal standard] was added. The samples were subsequently vortexed and mixed for 30 s, after which steel beads were added. The samples were then ground for 4 min at 45 Hz and sonicated for 5 min (in an ice-water bath). These steps were repeated 2–3 times. The samples were then incubated at −20°C for 1 h and centrifuged at 4°C and 12,000 rpm for 15 min. The supernatant was analyzed by using ultra-high-performance liquid chromatography (UHPLC)-QE Orbitrap/MS. An equal amount of supernatant was taken from all samples and mixed with QC samples for analysis. LC-MS/MS analysis: Analysis was performed using an UHPLC system (1,290, Agilent Technologies Inc., CA, United States) and a UPLC HSS T3 column (2.1 mm × 100 mm, 1.8 μm). The injection volume was 3 μL, and the instrument was operated in positive ion mode. Mobile phase A consisted of 0.1% formic acid aqueous solution, and mobile phase B was ACN. In negative ion mode, mobile phase A was 5 mmol L^–1^ ammonium acetate aqueous solution, and mobile phase B was ACN.

Data preprocessing and annotation: ProteoWizard software was used to convert the MS raw data into mzML format using ProteoWizard and processed by R package XCMS (version 3.2). OSI-SMMS (version 1.0, Chem Data Solution Information Technology Co., Ltd., Dalian, China) was used to identify the substances.

### Statistical analysis

Data processing and statistical analysis were performed by one-way analysis of variance (ANOVA) using SPSS 21.0 (SPSS, Inc., Chicago, IL, United States), and the significant difference between treatments was determined using Duncan’s multiple range test (*p* < 0.05).

Protein and metabolomics data analysis: Functional enrichment was performed using Gene Ontology (GO)^1^ (Dennis et al., 2003; Huang et al., 2009). The screening criteria for DEPs were a *P*-value < 0.05 and fold change <0.83 or >1.2. The screening criteria for DEMs were a *P*-value < 0.05 by Student’s *t*-test and a variable importance in projection (VIP) value of the first principal component of the orthogonal projections on latent structure-discriminant analysis (OPLS-DA) model greater than 1. The DEPs and DEPs were calculated using the “spearman” algorithm, the DEPs and DEMs were sorted and imported into the Kyoto Encyclopedia of Genes and Genomes (KEGG) database,^2^ and two omics datasets were analyzed by a combined analysis method.

## Results

### Rice plant growth

Compared with the S0 treatment, the SR treatment reduced rice plant height, root length, and the number of tillers and leaves (Supplementary Figure 1). The number of tillers and the shoot and root dry weights of rice in the S0 treatment were significantly higher than those in the SR treatment (Table 1). Compared with those in the S0 treatment, the tillers number, shoot weight, and root dry weight in the SR treatment were decreased by 25.0, 45.3, and 33.6%, respectively. The root/shoot ratio in the SR treatment was significantly higher than that in the S0 treatment and increased by 36.4%. Thus, the SR treatment severely inhibited the growth of rice.

**TABLE 1:** Effects of rice straw on rice plant growth.

Treatments	Tiller number per hole	Shoot dry weight (g hole^–1^)	Root dry weight (g hole^–1^)	Root-shoot ration
S0	12.00 ± 0.00a	3.44 ± 0.32a	1.51 ± 0.14a	0.44 ± 0.01b
SR	9.00 ± 0.00b	1.88 ± 0.14b	1.13 ± 0.11b	0.60 ± 0.02a

All the values are presented as the means ± SDs. The different lowercase letters indicate significant differences at *p* < 0.05 by Duncan’s multiple range test, longitudinal comparison.

### Chloroplast ultrastructure and non-structural carbohydrate contents of rice leaves

**FIGURE 1:**
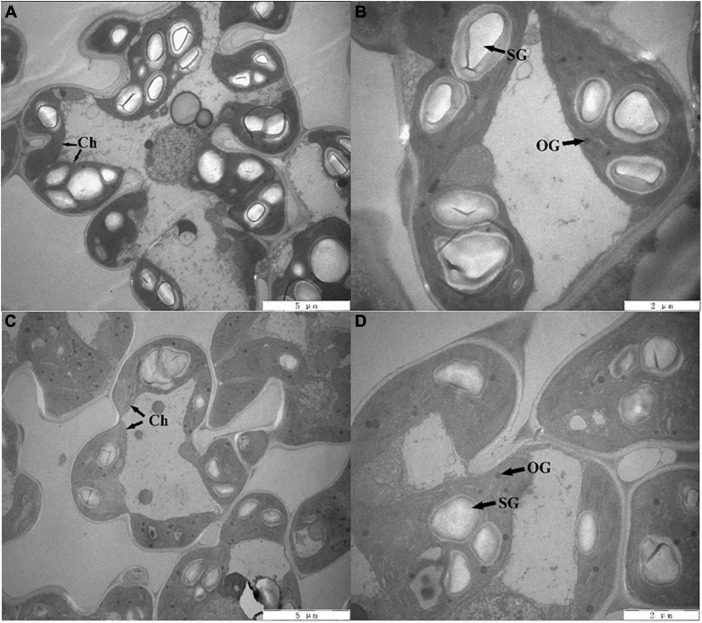
Chloroplast ultrastructure of rice leaves. Panels **(A,B)** represent no straw return (S0), panels **(C,D)** represent rice straw return (SR). Ch, chloroplast; OG, osmiophilic globules; SG, starch grains, **(A,C)** scale bar = 5 μm; **(B,D)** scale bar = 2 μm.

The changes in the chloroplast ultrastructure are shown visually in Figure 1. Under the S0 treatment, the size and number of starch grains were larger, with a typical arrangement of granum and stromal thylakoids, and the number of osmiophilic globules was small. The size of starch grains was decreased, the arrangement of stroma thylakoids became loose and disorderly, and the number of osmiophilic globules increased in the SR treatment compared with the S0 treatment. These results indicated that rice straw return inhibited the development of chloroplasts.

The contents of starch, sucrose, and soluble sugar significantly decreased in the SR treatment compared with the S0 treatment, decreasing by 10.0, 8.9, and 10.0%, respectively (Table 2). Rice straw return significantly decreased the non-structural carbohydrate contents of rice leaves.

**TABLE 2:** Non-structural carbohydrate contents of rice leaves (mg g^–1^ fresh weight).

Treatments	Starch	Sucrose	Soluble sugar content
S0	20.96 ± 0.37a	474.89 ± 2.39a	499.71 ± 6.03a
SR	18.87 ± 0.17b	433.21 ± 3.25b	449.89 ± 4.76b

All the values are presented as the means ± SDs. The different lowercase letters indicate significant differences at *p* < 0.05 by Duncan’s multiple range test, longitudinal comparison.

### Proteomics analysis of rice leaves

The proteomics changes between the SR and S0 treatments were analyzed using proteomic techniques. A total of 632 DEPs, namely, 329 upregulated and 303 downregulated proteins, were identified *via* LC–MS/MS for data acquisition with the criteria of a fold change (FC) >1.2 or <0.83 and a *P*-value < 0.05 (Table 3 and Supplementary Table 2).

**TABLE 3:** Statistics protein quantitative differences.

Comparisons	Upregulated proteins	Downregulated proteins	Total amounts
SR vs. S0	329	303	632

Rice straw return changed the abundance of proteins related to photosystem. In the SR treatment, chlorophyll a-b binding protein, photosystem I P700 chlorophyll a apoprotein, photosystem I reaction center subunit VI, photosystem I assembly protein, photosystem II D2 protein, photosystem II reaction center protein, cytochrome b6, cytochrome b6-f complex subunit, plastocyanin, and etcetera were downregulated. The downregulation of these proteins weakened the photosynthetic capacity in rice leaves. Superoxide dismutase (SOD), catalase (CAT), and glutathione S-transferase (GST) were downregulated, and peroxidase (POD) was upregulated in the SR treatment, which changed the ability to scavenge reactive oxygen species (ROS) in rice leaves. In addition, the abundance of carbon metabolism-related proteins was also changed, glyceraldehyde-3-phosphate dehydrogenase (GAPDH), 6-phosphofructokinase (PFK), fructose-bisphosphate aldolase (FBA), phosphoenolpyruvate/phosphate translocator, and etcetera were downregulated, which affected the carbon metabolism in rice leaves.

**FIGURE 2:**
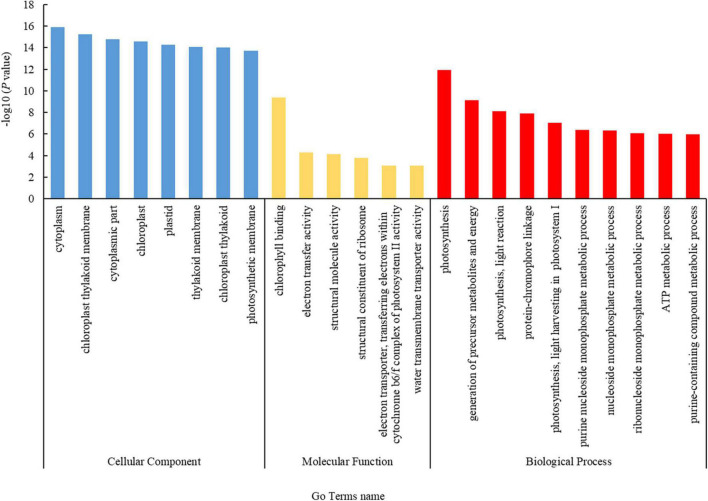
Gene Ontology (GO) annotation statistics of the differentially expressed proteins (DEPs). The GO annotations included cellular component, molecular function, and biological process terms, which are color coded as blue, yellow, and red, respectively. The ordinate indicates the negative common logarithm of the *P*-value derived from enrichment analysis, i.e., –log_10_ *P*-value, for each annotation.

Based on the functional terms, the DEPs were divided into three groups: those involved in cellular components (CCs), molecular functions (MFs), and biological processes (BPs) (Figure 2). In the CC group, 142 DEPs were mostly enriched in the cytoplasm (71.1%, 101 proteins), chloroplast thylakoid membrane (14.8%, 21 proteins), cytoplasmic part (62.0%, 88 proteins), chloroplast (30.3%, 43 proteins), plastid (30.3%, 43 proteins), and thylakoid membrane (14.8%, 21 proteins), among others. In the MF group, 142 DEPs were mostly enriched in chlorophyll binding (4.9%, 7 proteins), electron transfer activity (6.3%, 9 proteins), structural molecule activity (9.2%, 13 proteins), structural constituent of ribosome (7.7%, 11 proteins), electron transporter, transferring electrons within cytochrome b6/f complex of photosystem II (PSII) activity (1.4%, 2 proteins), and water transmembrane transporter activity (2.1%, 3 proteins). In the BP group, 141 DEPs were mostly enriched in photosynthesis (12.1%, 17 proteins), generation of precursor metabolites and energy (14.9%, 21 proteins), photosynthesis, light reactions (7.1%, 10 proteins), protein-chromophore linkage (5.0%, 7 proteins), photosynthesis, light harvesting in photosystem I (PSI) (3.5%, 5 proteins), purine nucleoside monophosphate metabolic process (7.8%, 11 proteins), nucleoside monophosphate metabolic process (8.5%, 12 proteins), ribonucleoside monophosphate metabolic process (7.8%, 11 proteins), ATP metabolic process (7.1%, 10 proteins), and purine-containing compound metabolic process (9.2%, 13 proteins).

**FIGURE 3:**
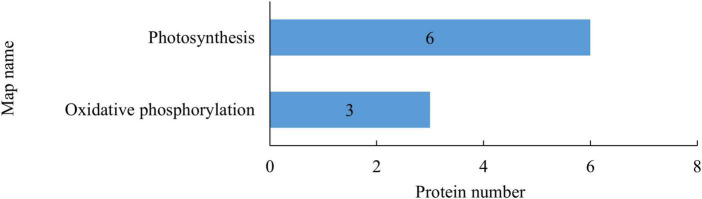
Kyoto Encyclopedia of Genes and Genomes (KEGG) pathways of differentially expressed proteins (DEPs) in rice leaves after straw return. The abscissa indicates the number of DEPs involved in the pathways, and the ordinate indicates the pathways in which the differentially expressed metabolites (DEMs) are involved.

Kyoto Encyclopedia of Genes and Genomes enrichment analysis was conducted to identify potential regulatory pathways that the DEPs may be involved in. As shown in Figure 3, the DEPs after rice straw return were mostly involved in photosynthesis (6 downregulated) and oxidative phosphorylation (1 upregulated, 2 downregulated). Rice straw return thus mainly affected the proteins involved in energy metabolism in leaves.

### Metabolomics analysis of rice leaves

The changes in the metabolic profiles of the rice leaves between the SR and S0 treatments were analyzed using the metabolomics method based on HILIC UHPLC-Q-TOF technology. A total of 115 DEMs were identified, namely, 44 upregulated and 71 downregulated DEMs, with an OPLS-DA model VIP > 1 and a *P*-value < 0.05 used as criteria (Supplementary Table 3).

**FIGURE 4:**
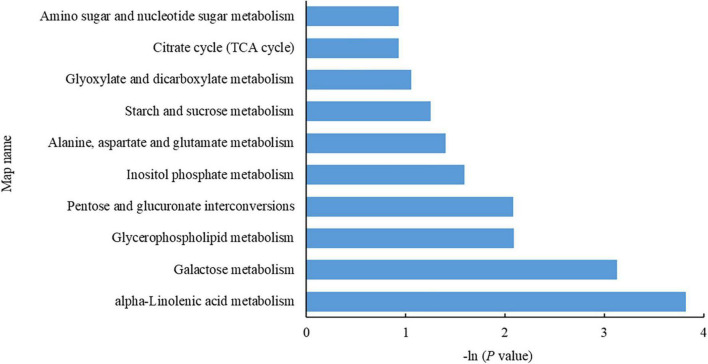
Metabolic pathways enriched with differentially expressed metabolites (DEMs). The abscissa indicates a negative common logarithm of the *P*-value derived from enrichment analysis, i.e., –ln (*P*-value); the ordinate indicates the pathways in which the DEMs are involved.

As shown in Figure 4, the DEMs were enriched mostly in alpha-linolenic acid metabolism (2 downregulated), galactose metabolism (1 upregulated, 1 downregulated), glycerophospholipid metabolism (1 upregulated, 1 downregulated), pentose and glucuronate interconversion (1 upregulated), inositol phosphate metabolism (1 downregulated), alanine, aspartate and glutamate metabolism (1 upregulated), starch and sucrose metabolism (1 upregulated), glutathione metabolism (1 upregulated), glyoxylate and dicarboxylate metabolism (1 upregulated), the TCA cycle (1 upregulated), and amino sugar and nucleotide sugar metabolism (1 upregulated).

### Integrated proteomic and metabonomic analysis

**FIGURE 5:**
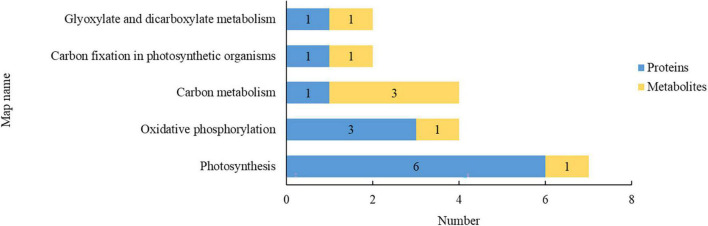
Pathways involving both differentially expressed proteins (DEPs) and differentially expressed metabolites (DEMs). Blue and yellow indicate the number of DEPs and DEMs involved, respectively.

The proteomics and metabolomics were integrated to analyze the relationship between proteins and metabolites, and the DEPs and DEMs were compared with the KEGG database. The 5 KEGG pathways involving both DEPs and DEMs are presented in Figure 5. In terms of the number of DEPs and DEMs involved, the pathways were in the descending order of photosynthesis, oxidative phosphorylation, carbon metabolism, carbon fixation in photosynthetic organisms, and glyoxylate and dicarboxylate metabolism. Compared with the S0 treatment, the SR treatment resulted in DEPs and DEMs that were mostly associated with pathways of carbohydrate metabolism and energy metabolism, indicating that rice straw return mainly affected carbohydrate metabolism and energy metabolism in rice leaves.

## Discussion

### Effect of straw return on rice growth

Rice straw return enhances more reducing conditions in paddy soils, the redox status was strongest at 3–6 weeks after straw return, and the tillers number and plant height of rice were significantly reduced at the 4th week after straw return (Tanji et al., 2003; Gao et al., 2004). In this study, the tillers and dry weight of rice plants were significantly decreased in the SR treatment (Table 1), which may be due to the straw return promotes reducing conditions in the soil, resulting in the accumulation of harmful substances (Gao et al., 2004; Shan et al., 2008), and inhibits the growth of rice plants. Drought stress induced the suppression of both shoot and root growth, and shoot growth was suppressed more severely than root growth (Yamane et al., 2003a). In this study, the root/shoot ratio significantly increased in the SR treatment compared with the S0 treatment, which may be because rice supplied more energy for root growth to adapt to the adverse conditions caused by straw return.

Under drought stress, chloroplasts in rice leaves were inflated, devoid of starch grains and were displaced from the cell wall, and the chloroplast envelope was destroyed (Yamane et al., 2003a; Gayen et al., 2019). Yamane et al. (2003b) investigated the ultrastructure of chloroplasts in salt-treated rice seedlings under NaCl and polyethylene glycol (PEG) stress. NaCl induced swelling of thylakoids and caused only slight destruction of the chloroplast envelope, and PEG stress did not induce thylakoids to swell but did cause severe destruction of the chloroplast envelope. In the SR treatment, the arrangement of stromal thylakoids became loose and disorderly, the size of starch grains decreased, and the number of osmiophilic globules increased (Figure 1). The structure of thylakoids affects leaf photosynthetic capacity (Jiang et al., 2011). Carbohydrate energy generated by plants through photosynthesis is used as substrates for growth, or stored as reserves, and carbohydrate metabolism regulates plant growth (Eveland and Jackson, 2012). The ultrastructure of chloroplasts was destroyed in the SR treatment, which reduced the photosynthetic capacity of leaves, and the contents of sucrose, starch, and soluble sugars decreased significantly (Table 2); thus, the supply of carbohydrates for growth and energy storage was insufficient.

### Effects of straw return on the energy metabolism of rice leaves

Photosynthesis is a well-established source of ROS in plants, and light-driven ROS production has the potential to cause irreversible damage to photosynthetic components (Foyer and Shigeoka, 2011). To scavenge ROS, cells have evolved a system of enzymatic and non-enzymatic antioxidants such as SOD, CAT, GST, and phenolic compounds (Ahmad et al., 2010; Komatsu et al., 2014). Antioxidative enzymes such as SOD, CAT, and ascorbate peroxidase (APX) and non-enzymatic scavengers decreased in plants under drought stress (Impa et al., 2012). Similarly, POD activity in rice roots was shown to strongly increase in response to high-salinity stress. In this study, SOD, CAT, and GST expression levels were downregulated in the SR treatment (Supplementary Table 2), which reduced the ability to scavenge ROS and caused damage to photosynthetic components, decreased the photosynthetic capacity of leaves. However, POD expression was upregulated in the SR treatment, which was similar to the results of Cunha et al. (2016).

We found that most of the proteins involved in photosynthesis were changed in the SR treatment. The main function of light-harvesting chlorophyll a/b-binding protein is to collect and transfer light energy to photosynthetic centers (Xia et al., 2012), which is the most sensitive to climate change in disturbed ecosystems (Gayen et al., 2019). The chlorophyll a-b binding proteins were downregulated in the SR treatment, which reduced the light energy transfer in photosynthesis and weakened the photosynthetic capacity. ATP synthase decreases with water stress (Tezara et al., 1999), the SR treatment decreased ATP synthase, reducing the amounts of ATP, and the photosynthetic assimilation CO_2_ was limited.

**FIGURE 6:**
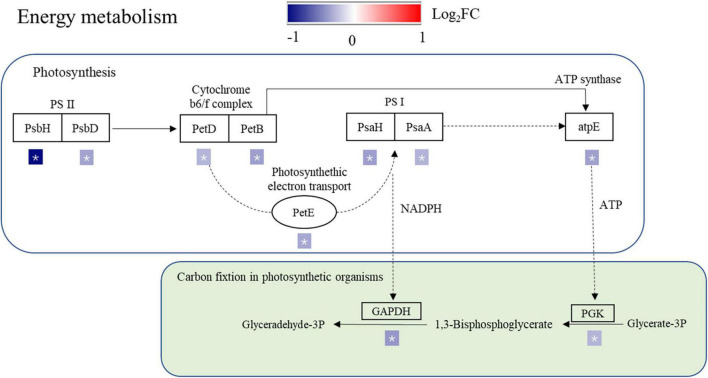
Differentially expressed proteins (DEPs) associated with the photosynthesis pathway and carbon fixation in the photosynthetic organism pathway. PsbH, PSII reaction center protein H; PsbD, PSII D2 protein; PetD, cytochrome b6-f complex subunit 4; PetB, cytochrome b6; PetE, plastocyanin; PsaH, photosystem I reaction center subunit VI; PsaA, PSI P700 chlorophyll a apoprotein A1; atpE, ATP synthase epsilon chain; PGK, phosphoglycerate kinase; GAPDH, glyceraldehyde-3-phosphate dehydrogenase; FBA1, fructose-bisphosphate aldolase. The different protein changes are represented by log_2_(FC). Red and blue indicate upregulated and downregulated proteins, respectively, * indicates a significant difference.

Photosystem I is the second photosystem in the photosynthetic light reaction complexes of plants, which uses light energy to produce the high-energy carrier NADPH (Amunts et al., 2007). Electron transport between the Cyt b6/f complex and PSI is performed exclusively by PetE (Ivanov et al., 2012). Excessive reduction of the electron transport chain under drought stress results in the production of ROS in plant mitochondria and chloroplasts (Ahmad et al., 2014). The downregulation of PetE in the SR treatment resulted in a decrease in electron transport, which in turn affected the synthesis of NADPH and ATP (Figure 6), the decreased electron transport may cause an increase in ROS production. The levels of photosynthetic proteins and the activity of PSII were shown to be reduced in rice under salt stress (Tiwari et al., 1998; Xu et al., 2015). In this study, PsbH, PsbD, PetD, PetB, PetE, PsaH, PsaA, and atpE expression levels were downregulated in the SR treatment (Figure 6), which reduced the production of NADPH and ATP, reducing the energy supply for carbon fixation in photosynthetic organs. Phosphoglycerate kinase is a key component of ATP production in the Calvin cycle for carbon fixation in plants. Under abiotic stress conditions, the expression levels of phosphoglycerate kinase (PGK), FBA, and PSII oxygen-evolving complex protein 1 precursor were downregulated under salt stress (Caruso et al., 2008). Stress conditions decreased oxidized NADP^+^, which acts as an electron acceptor in photosynthesis (Ahmad et al., 2010). In this study, PGK and GAPDH expression levels were downregulated in the SR treatment (Figure 6), and carbon fixation was limited in rice leaves, leading to a reduction in NADP^+^ production, and a decrease in the synthesis and transport of photosynthates, which would further affect carbon metabolism in rice leaves. In addition, the synergistic downregulation of proteins involved in photosynthesis and the Calvin cycle under stress, this may be a defense strategy to avoid the overproduction of ROS (Komatsu et al., 2014) and reduce the damage of straw return to rice growth.

### Effect of straw return on carbon metabolism in rice leaves

**FIGURE 7:**
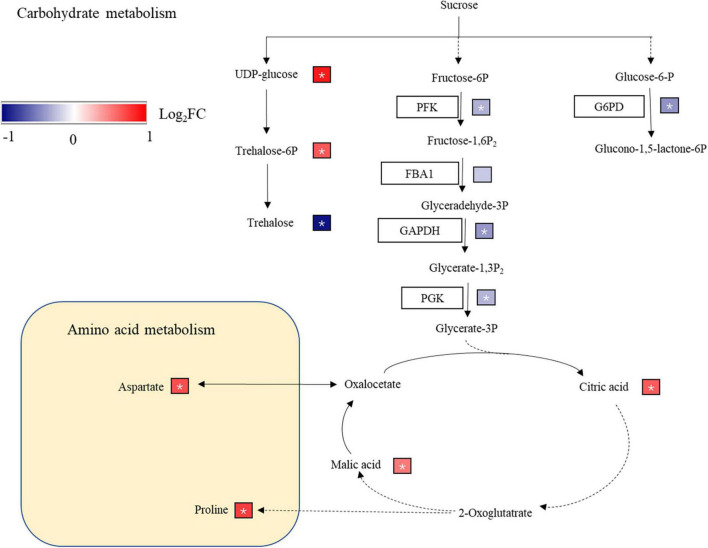
Carbon metabolism and amino acid biosynthesis pathway. G6PD, glucose-6-phosphate 1-dehydrogenase; PFK, 6-phosphofructokinase; FBA1, fructose-bisphosphate aldolase; GAPDH, glyceraldehyde-3-phosphate dehydrogenase; PGK, phosphoglycerate kinase; GOT1, aspartate aminotransferase; mdh, malate dehydrogenase. The different protein expression levels changes were represented by log_2_(FC) in expression. Red and blue indicate upregulated and downregulated proteins, respectively,* indicates a significant difference.

Sugar is the fuel of cell metabolism and an important signal molecule (Rolland et al., 2006). Under abiotic stresses where sugar availability is low, sugar-derived signaling systems can modulate nutrient, energy signaling and metabolic processes (Liu et al., 2013). Sucrose and trehalose 6-phosphate (T6P) can regulate the growth, development and metabolism of plants, especially under abiotic stress (Paul et al., 2008; Eveland and Jackson, 2012). T6P accumulation in *Arabidopsis* seedlings inhibited the growth of seedlings, and T6P control over carbon utilization was related to available carbon for growth (Schluepmann et al., 2004). In the rice leaves of the SR treatment (Figure 7), the non-structural carbohydrate content was significantly decreased, and T6P expression was upregulated. When T6P and carbon availability were unbalanced, T6P accumulation may be one of the reasons for inhibiting the growth of rice. Trehalose interferes with carbon allocation to the roots by inducing starch synthesis in the shoots of *Arabidopsis* (Wingler et al., 2000). The trehalose content was decreased in the SR treatment (Figure 7), which may reduce the starch content in leaves, distribute more carbohydrates to roots, and increase the root/shoot ratio of rice.

Glyceraldehyde-3-phosphate dehydrogenase is essential to glycolysis and may regulates the plant response to abiotic stress (Hancock et al., 2005). The expression of GAPDH was downregulated in wheat seedlings after 3 days of salt stress (Kamal et al., 2012), and the upregulation of GAPDH enhances the salt tolerance of rice seedlings (Li et al., 2010). The expression levels of PFK, GAPDH, and PGK, which are involved in the glycolysis pathway, were downregulated in the SR treatment (Figure 7), which reduced the production of ATP in rice leaves and energy supply for rice growth. GAPDH expression was downregulated in the SR treatment (Figure 7), which reduced the tolerance of rice to stress. Glucose-6-phosphate (G6PD) is the rate-limiting enzyme in the pentose phosphate pathway (Bolouri-Moghaddam et al., 2010). The downregulation of G6PD expression in SR treatment inhibited the metabolism of the pentose phosphate pathway, reduced the energy supply and production of anabolic materials in plants (Figure 7). The SR treatment reduced carbohydrate metabolism and energy supply, these may inhibit the rice growth.

Plants accumulate soluble sugars to tolerate osmotic stress, which protects plants from abiotic stress (Chinnusamy et al., 2006). Under salt stress, a large amount of sugars and amino acids accumulate in rice plants, and organic acids increase in rice roots (Zhao et al., 2014; Wang et al., 2016). Organic acids (malic acid, citric acid, etc.) accumulate under drought stress and play an important role in the osmotic regulation of plants (Franco et al., 1992; Hummel et al., 2010). In the SR treatment, galactose, trehalose, and maltotriose were downregulated, possibly because straw return reduced the photosynthesis of rice leaves, and decreased the synthesis ability of carbohydrates; thus, the content of soluble sugar decreased. At the same time, citric acid, and malic acid were upregulated (Supplementary Table 3), helping maintain the osmotic pressure of cells.

Ribosomal proteins play a crucial role in protein synthesis (Kim et al., 2014b). In the SR treatment, 40S ribosomal protein S21/S8/S26, and 50S ribosomal protein L36/L33 were upregulated, while 40S ribosomal protein S27/S13-2, and 50S ribosomal protein L35/L14 were downregulated. The change of ribosomal proteins may affect protein synthesis. The accumulation of amino acids in plants helps maintain the stability of membrane and protein structures under lower osmotic potential. Proline plays an important role in osmotic regulation and subcellular structure protection under abiotic stress (Reddy et al., 2004; Ashraf and Foolad, 2007) and acts as part of signal transduction pathways that regulate stress-responsive genes. Proline accumulates in plants under salt and drought stress conditions (Khedr et al., 2003; Gayen et al., 2019). Aspartate is a precursor amino acid for the synthesis of methionine, and ROS can oxidize methionine in plant cells (Azevedo et al., 1997; Hesse and Hoefgen, 2003; Zhao et al., 2022). Proline, aspartate, and methionine contents were increased in the SR treatment (Supplementary Table 3), which helped maintain cell osmotic pressure, the stability of membrane and protein structures, protected subcellular structure, and enhanced the ability to defend against oxidative stress.

Glutathione can detoxify ROS and protect plants from oxidative damage (Waśkiewicz et al., 2014). Flavonoids stabilize the membrane through a decrease in lipid fluidity, sterically hinder the diffusion of free radicals, and inhibit membrane peroxidation (Arora et al., 2000). In the SR treatment, the upregulation of glutathione and flavonoids expression helped to maintain the osmotic pressure of cells, scavenged free radicals, and enhanced the resistance of rice plants to straw return. Aquaporins are present in the plasma membrane and vacuoles and facilitate the diffusion of water and neutral solutes across the cell membrane (Maurel and Chrispeels, 2001; Vera-Estrella et al., 2004). The downregulation of aquaporin (TIP1-1/PIP2-6/TIP1-2) expression in the SR treatment reduced the water transport capacity in rice leaf cells. Heat shock protein 70 (HPS 70) regulates apoptosis (Beere and Green, 2001) and plays an important role in protein folding under adverse environmental conditions (Fu et al., 2012). Under salt stress, HPS 70 and ATP synthase CF1 β-chain significantly increased in rice seedlings (Kim et al., 2005). Salt stress downregulates S-adenosylmethionine synthetase in the roots of rice seedlings (Li et al., 2010), and S-adenosylmethionine synthetase probably enhances the salt tolerance of rice seedlings. In the SR treatment, HPS 70 kDa (LOC_Os12g14070) expression levels were upregulated (Supplementary Table 2), which may reduce apoptosis, S-adenosylmethionine synthase expression was downregulated (Supplementary Table 2), similar to that under salt stress. Linoleate and linolenate are the major fatty acids in plant membranes, and free linoleate and linolenate are precursors for the synthesis of jasmonic acid and oxidized lipids which are essential components for the plant response to abiotic stress (Weber, 2002). Linoleate and linolenate contents were decreased in the SR treatment (Supplementary Table 3), indicating that SR treatment affected the structure of the plant membrane and reduced resistance to abiotic stress. Notably, in this study, the changes in DEPs and DEMs in the straw return treatment were mostly similar to those under drought and salt stress, but there were also differences. It may be that the degradation of straw altered the soil environment and caused osmotic stress to rice seedlings, which was not conducive to rice growth. The specific reasons remain to be further studied.

## Conclusion

Straw return inhibited rice growth and destroyed the chloroplast ultrastructure in rice leaves. Compared with no straw return, straw return mainly affected photosynthesis, carbon fixation in photosynthetic organs, glycolysis, the TCA cycle, etc. Straw return downregulated the expression of proteins involved in energy metabolism and carbohydrate metabolism in rice leaves, resulting in a reduction in the substances and energy required for rice growth. Organic acids, amino acids, and other osmotic substances accumulated in the plants in the straw return treatment, enhancing the osmotic regulatory ability of the rice leaves.

## Data availability statement

The data presented in this study are deposited in the ProteomeXchange repository, accession number: PXD035835.

## Author contributions

SY designed the experiment. SY, JL, and JF performed the material preparation and data collection. SG, KS, and HZ analyzed the data. SY wrote the manuscript with support from ZG and ZZ. CY supervised and complemented the writing of the manuscript. All authors read and approved the final manuscript.

## Footnotes

1
http://www.geneontology.org/

2
http://www.kegg.jp/

## Supplementary material

Supplementary Figure 1 Morphology of rice plant.
