# Supplementary figures and images for: Integrated proteomics and metabolomics analysis of rice leaves in response to rice straw return
Source: Front Plant Sci. 2022 Sep 13;13:997557. doi: 10.3389/fpls.2022.997557 (PMC9514043; doi:10.3389/fpls.2022.997557)

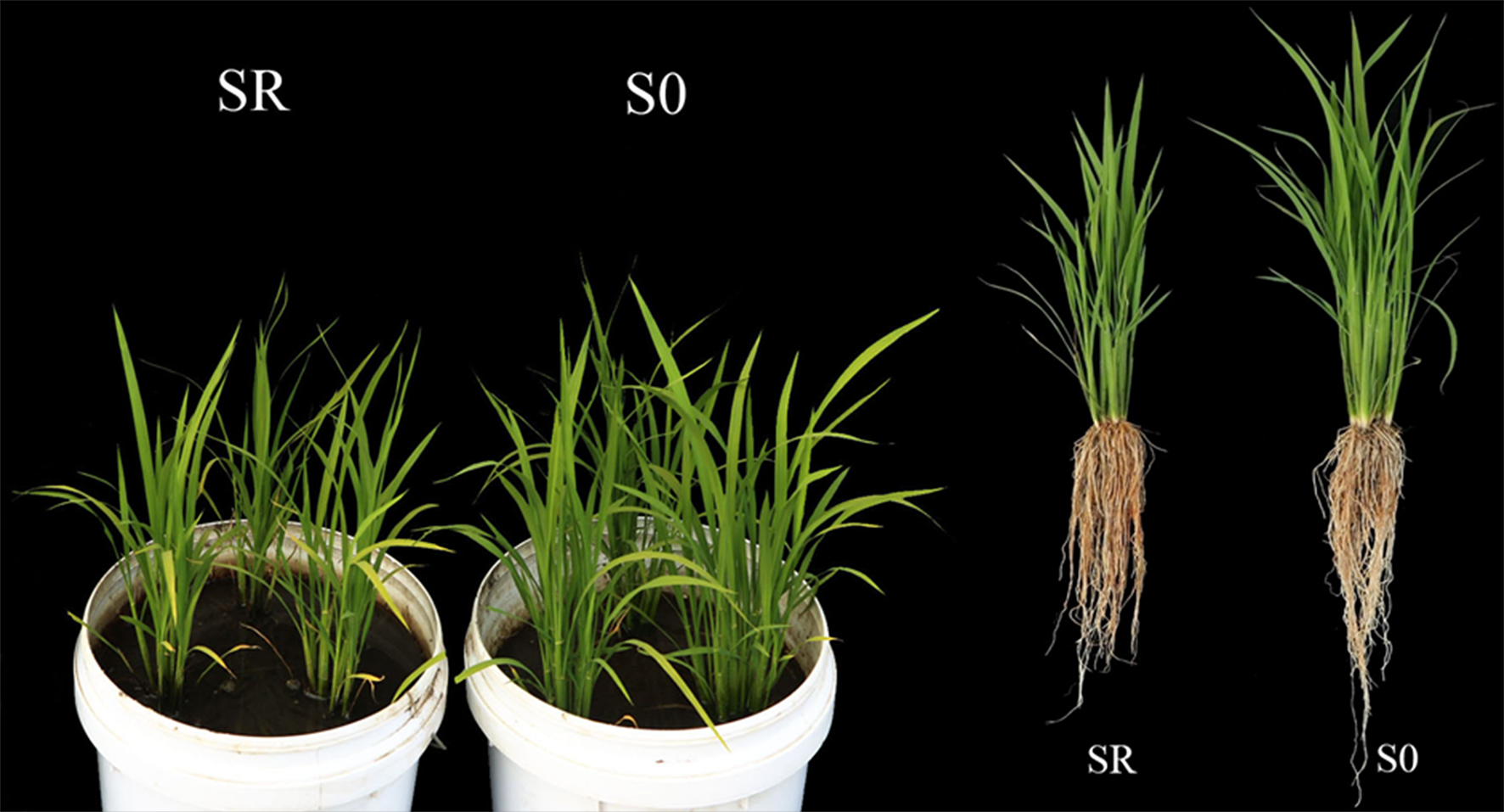

Supplement: Supplementary Figure 1 — Morphology of rice plant. [file Image_1.TIF]
